# Impact of radiotherapy on the prognosis in uterine cervical adenocarcinoma: a meta-analysis and retrospective cohort study

**DOI:** 10.3389/fonc.2025.1653107

**Published:** 2025-09-09

**Authors:** Keyi Zhang, Jianan Ji, Jing Yang, Shulin Zhou, Jiangnan Qiu, Chengyan Luo

**Affiliations:** ^1^ Department of Gynecology, Fujian Medical University Affiliated Zhangzhou Hospital, Zhangzhou, China; ^2^ Department of Gynecology, First Affiliated Hospital with Nanjing Medical University, Nanjing, China

**Keywords:** uterine cervical adenocarcinoma, radiotherapy, overall survival, disease-free survival, cancer-specific survival

## Abstract

**Introduction:**

The treatment of uterine cervical adenocarcinoma (UAC) has always been a clinical challenge. The study investigated the effect of radiotherapy (RT) on survival outcomes and tumor recurrence in patients with UAC and further explored potential candidates who may benefit from RT.

**Methods:**

We systematically searched the literature on the effects of RT on disease-free survival (DFS) and overall survival (OS) for UAC and performed a meta-analysis. The impact of RT on the cancer-specific survival (CSS) and DFS were retrospectively analyzed with the UAC cases from the Surveillance, Epidemiology, and End Results (SEER) database and at our center. A total of 4382 patients from the SEER database after propensity score matching and 315 cases at our center were retrieved, respectively. Univariate and multivariate Cox regression analysis were employed to investigate the effect of RT on the prognosis. Subgroup analysis was conducted to identify populations that may benefit from RT.

**Results:**

The meta-analysis revealed that RT didn’t improve DFS (OR: 0.72, 95% CI 0.34 - 1.52) and OS (OR: 0.44, 95% CI 0.18 - 1.07), in FIGO stage IB-IIA patients with UAC. The retrospective study found that RT improved CSS (HR: 0.87, 95% CI 0.78 - 0.9), but not DFS (HR: 2.62, 95% CI 0.96 - 6.8). Those with stage pT2-4, pN1, pM1, primary tumor size > 39 mm, grade III-IV, or unresected primary tumors had superior CSS when treated with RT than those without RT. In terms of DFS, the cases staged as pT1-2, pN0, pM0, with tumor > 19 mm, graded III-IV, resection of primary tumor, no parametrial involvement, and with or without lymph-vascular stromal invasion had worse outcomes after receiving RT.

**Conclusion:**

The UAC patients with more advanced, larger primary tumors, higher histological grade, and unresected primary tumors are likely to benefit from RT, which need to be substantiated by prospective studies.

## 1 Introduction

Cervical cancer (CC) is the fourth most common and cause of death amongst all female malignancies worldwide (1). Uterine cervical adenocarcinoma (UAC) is one of the frequent histologic types of CC (2, 3), accounting for about 20% of cases, just after uterine cervical squamous cell carcinoma (USC). The prevalence and mortality of UAC have been on the rise, and the incidence tends to be younger (4, 5).

UAC covers a wide range of histologic subtypes and is characterized by a significant heterogeneity of biological behavior, leading to a diverse prognosis (6, 7). Previous studies have indicated that UAC is more aggressive (8) and less sensitive to radiotherapy (RT) and chemotherapy (ChT) than USC (9, 10), making its treatment more challenging. Cong et al. revealed that patients with International Federation of Gynecology and Obstetrics (FIGO) 2009 stage IA-IIA2 UAC were more likely to develop uterine corpus infiltration, lymph node, ovarian transference, and peritoneal metastases compared with USC, and suffered higher rates of cancer-related death and disease progression (9). A study by Liu et al. suggested that in FIGO 2018 stage IIB-IV CC patients, UAC patients had significantly lower 5-year disease-free survival (DFS) and overall survival (OS) than USC patients, even after receiving the same radical RT (10). Currently, there is a lack of guidelines unique to UAC, and the treatment of UAC in clinical practice is mainly referred to the USC criteria. Therefore, the optimal treatment of UAC needs to be further explored.

RT plays an important role in the treatment of CC, including concurrent chemoradiotherapy (CCRT) and adjuvant RT (aRT). However, the available studies have inconsistent results regarding the sensitivity of UAC to RT (11, 12). Kazuhiro et al. (11) conducted a retrospective study of 76 UAC patients with pelvic lymph node metastases and showed that aRT after radical hysterectomy did not improve OS. A retrospective analysis of UAC patients using the SEER database found that RT produced improved OS in patients without distant metastases and poorer OS in those with distant metastases (12). However, the study did not analyze the effect of confounding factors such as primary tumor stage, surgery, and ChT on this finding.

In addition to the sensitivity of UAC to RT, both RT-related toxicity, involving lower gastrointestinal tract, bladder and vagina, and their impact on quality of life need to be considered in clinical decision-making. Previous studies have reported the incidence of rectal toxicity ranging from 29.7% to 40% and bladder toxicity ranging from 21.8% to 28% in patients with CC receiving definitive RT (13). A meta-analysis by Raj et al. (14) showed that the overall incidence of vaginal toxicity in patients with CC after definitive CCRT was 39% (95% CI: 21 - 56%), with vaginal stenosis being the most common toxic response (15). These patients present with bowel bleeding, urinary incontinence, urinary frequency, and hematuria, necessitating hospital visits (16, 17), thus increasing the psychological and financial burdens.

Accordingly, whether RT can provide benefits to patients with UAC is a clinical concern. The study intends to evaluate the effect of RT on survival outcomes and tumor recurrence in patients with UAC using previous literatures, populations from various centers, and to further explore potential candidates who may benefit from RT.

## 2 Materials and methods

### 2.1 Search strategy, eligibility criteria and assessment of risk of bias for meta-analysis

Pubmed, Embase, Cochrane, China Biology Medicine (CBM), and China National Knowledge Infrastructure (CNKI) databases, as well as the WHO International Clinical Trials Registry Platform (ICTRP) and ClinicalTrials.gov websites, were searched from 1990 to June 2024 for literatures on RT in UAC patients to perform a meta-analysis. The search strategy is shown in **Table 1**. We included randomized controlled trials (RCTs), cohort studies, or case-control studies in which the diagnosis of UAC was confirmed pathologically and the treatment was divided into non-radiotherapy (NRT) and RT groups, with 5-year overall survival (OS) rates and 5-year disease-free survival (DFS) rates reported. Exclusion criteria included duplicate literatures, literatures with moderate to high bias in methods or reporting, reviews, case reports, or studies that lacked extractable or transformable data for analysis. The 2020 Preferred Reporting Items for Systematic Reviews and Meta-Analyses (PRISMA) flowchart (18) shows literature screening process for this review (**Supplementary Material Figure S1A**). The quality of each study was assessed using the Newcastle-Ottawa Scale (NOS) (19) and the 2019 Cochrane Risk-of-Bias tool (RoB2) (20) for non-RCTs and RCTs respectively. The literatures were included in this meta-analysis only when non-RCTs studies scored 7 stars or when the risk of bias for RCTs was low. Three researchers extracted the following data independently, and cross-checked them: number of all-cause deaths and recurrences among patients undergoing RT or NRT, number of participants in the RT and NRT group, RT modality, tumor stage, year of publishments. Where disagreements were noted, the researchers resolved them through discussion. The study protocol is registered in International prospective resister of systematic reviews (PROSPERO) under the number “CRD42018111659”.

**Table 1:** Retrieval strategy for meta-analysis.

Database	Retrieval statement
Pubmed	((“adenocarcinoma”[MeSH Terms] OR “adenocarcinoma”[Title/Abstract]) AND (“cervix uteri”[MeSH Terms] OR “cervical”[Title/Abstract] OR “cervix uter*”[Title/Abstract] OR “uterine cervix”[Title/Abstract] OR “uterine cervical neoplasms”[MeSH Terms])) AND (“Radiotherapy”[Mesh] OR “radiotherapy”[Title/Abstract] OR “radiation”[Title/Abstract])
Embase	(‘cervical adenocarcinoma’:ti,ab,kw OR ‘uterine cervix adenocarcinoma’/exp OR ‘uterine cervix adenocarcinoma’:ti,ab,kw) AND (radiation:ti,ab,kw OR radiotherapy:ti,ab,kw OR ‘uterine cervix adenocarcinoma’/exp)
Cochrane	ID Search Hits
	#1 MeSH descriptor: [Adenocarcinoma] explode all trees
	#2 MeSH descriptor: [Cervix Uteri] explode all trees
	#3 (cervical adenocarcinoma):ti,ab,kw OR (adenocarcinoma of cevix uteri*):ti,ab,kw OR (adenocarcinoma of cevix uteri*):ti,ab,kw
	#4 (radiotherapy):ti,ab,kw OR (radiation):ti,ab,kw
	#5 MeSH descriptor: [Radiotherapy] explode all trees
	#6 ((#1 AND #2) OR #3) AND (#4 OR #5) in Trials
CNKI	(篇关摘:宫颈腺癌)AND(篇关摘:放射疗法)^1^
CBM	(“宫颈腺癌”[全部字段:智能] AND “放射疗法”[全部字段:智能])^2^

CNKI, China National Knowledge Infrastructure; CBM, China Biology Medicine.

^1^(cervical adenocarcinoma):ti,ab,kw AND (radiotherapy):ti,ab,kw.

^2^(cervical adenocarcinoma) [all fileds] AND (radiotherapy)[all fileds]

### 2.2 Patient population, data acquisition and analysis

In this study, patients with pathologically confirmed primary UAC between January 2000 and December 2019 were retrieved from the U.S. Surveillance, Epidemiology, and End Results (SEER) database (21) using SEER*Stat 8.4.1 software (22) (www.seer.cancer.gov). The flowchart of data processing was shown in **Supplementary Material Figure S1B**. Due to the lack of information on lymph-vascular space invasion (LVSI), depth of tumor infiltration, vaginal margin status, parametrial infiltration (PI) status, and tumor recurrence in UAC patients included in the SEER database, we concomitantly collected data on patients pathologically diagnosed with primary UAC at the First Affiliated Hospital of Nanjing Medical University between January 2010 and September 2023 to explore the role of the above factors in the impact of RT on tumor recurrence (**Supplementary Material Figure S1C**). The included cases were pathologically diagnosed according to the International Classification of Diseases of Oncology, Third Edition (ICD-O-3) (23), had a primary site of the cervix (C53.0, C53.1, C53.8, and C53.9), and the histologic type of adenocarcinoma (8140/3, 8144/3, 8147/3, 8200/3, 8210/3, 8245/3, 8260/3, 8261/3, 8262/3, 8263/3, 8310/3, 8313/3, 8323/3, 8380/3, 8382/3, 8384/3, 8441/3, 8460/3, 8461/3), with definitive staging, treatment details, and complete follow-up information. The exclusion criteria were as follows: patients with cervical malignancies other than adenocarcinoma, with mixed cervical cancer containing adenocarcinoma, with metastatic cervical adenocarcinoma, with benign tumors of the cervix, with unknown stage, and with less than 1 month of follow-up. These patients were classified into RT and NRT groups based on their treatment included RT or not. The outcomes included cancer-specific survival (CSS) and DFS, where CSS time was defined as the time from diagnosis to death due to UAC or to the last follow-up, and DFS time was defined as the time from the end of treatment to UAC recurrence or to the last follow-up. The cases in our center were followed up until December 31, 2023. Two continuous variables were converted into categorical variables by combining thresholds obtained using the X-tile software (Yale University, New Haven, CT, USA) (24) and clinical significance: age was classified as ≤ 49, 50 - 69, and ≥ 70 years, and tumor size was classified as ≤ 19 mm, 20 – 39 mm, and ≥ 40 mm. In our center, only 7 patients (2.2%) were ≥ 70 years of age and therefore these patients were classified as ≤ 49-year-olds and > 49-year-olds. The study was performed according to the Declaration of Helsinki (as revised in 2013) (25). The SEER database is a public database that can be accessed by applying to the official website with no requirement for patients’ informed consent or institutional ethical approval. The section of studies sourced from the SEER database is not subject to patients’ informed consent and institutional ethical approval since it is a public database. The population-based studies included from our center was approved by the Ethics Committee of the First Affiliated Hospital with Nanjing Medical University (No. 2024-SR-261), with informed consent from the participants.

### 2.3 Statistical analysis

The study was statistically analyzed using SPSS software, version 27.0 (IBM Corp., Armonk, NY, USA), R software, version 4.2.2 (https://www.r-project.org/), and Stata SE version 16 (Stata Corp, College Station, TX, USA). The pooled odds ratio (OR) estimates were calculated using a random-effects model with the restricted maximum likelihood (REML) for OS and DFS. The heterogeneity among studies were assessed by the I^2^ and H^2^ statistic. Significant heterogeneity (I^2^ > 50% or H^2^ > 1.5) requires sensitivity analysis, which means recalculating the overall effect estimate after omitting each study. As fewer than ten studies were included, publication bias was not accessed in the statistics. In testing the significance of differences between groups, the Mann-Whitney U test was employed for the quantitative variables, the chi-square test for the qualitative variables. To balance baseline characteristics and improve comparability between the RT and NRT groups, we performed 1:1 propensity score matching (PSM) on the variables, using calipers of width equal to 0.01. To prevent overfitting of the model, LASSO (last absolute shrinkage and selection operator) regression analysis was employed to screen the variables. The CSS and DFS were estimated using the Kaplan-Meier method, and the differences between groups were tested with the log-rank test. Univariate and multivariate Cox proportional hazard models were employed to independent prognostic factors affecting CSS and DFS in patients with UAC. To explore the patients with UAC who would benefit from RT, we performed subgroup analyses. Differences were considered statistically significant at P values less than 0.05.

## 3 Results

### 3.1 The effect of RT on OS and DFS for patients with UAC in meta-analysis

**Figure 1:**
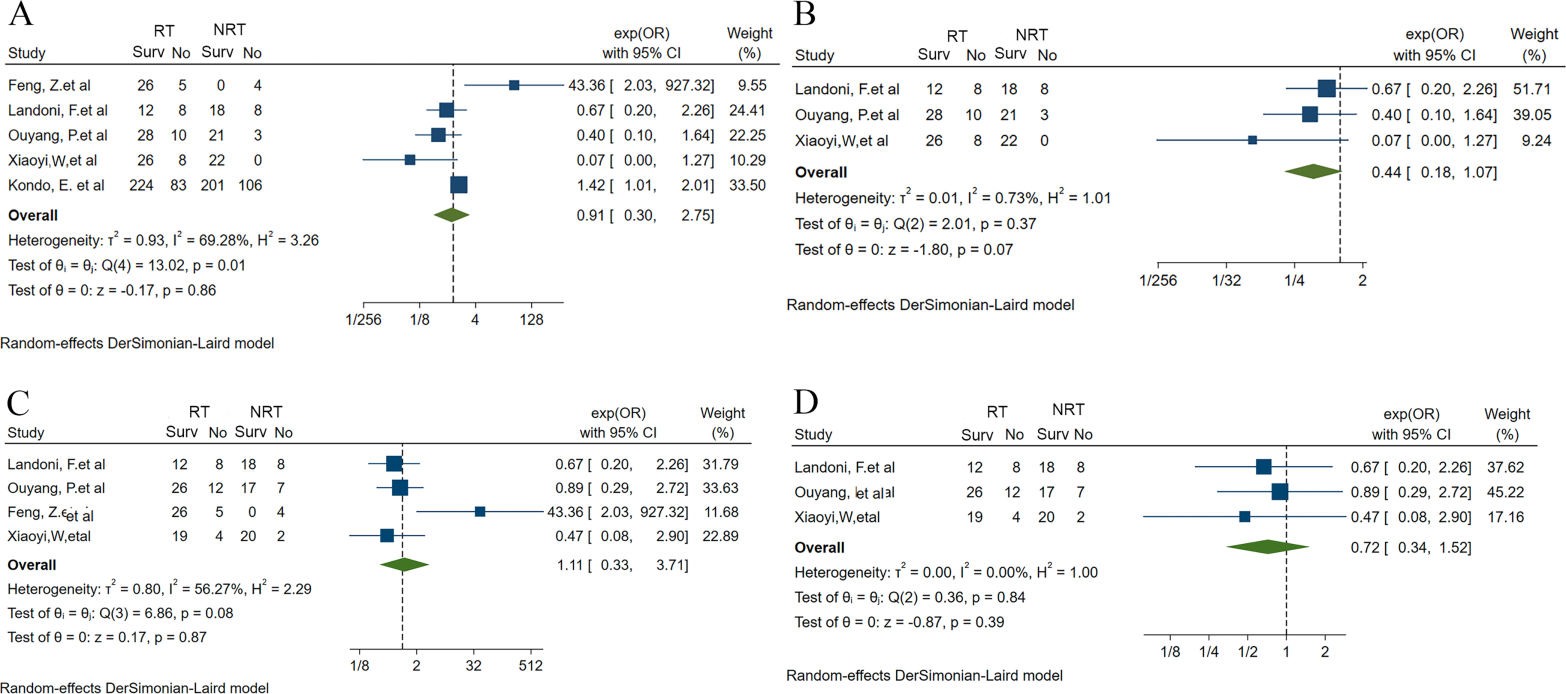
Forest plots of meta-analysis fraction for the impact of RT on the UAC patient’s prognosis (26–30). **(A)** 5-year OS rate for RT vs. NRT before sensitivity analysis. **(B)** 5-year OS rate for RT vs. NRT after sensitivity analysis and excluding the study that brought about heterogeneity. **(C)** 5-year DFS rate for RT vs. NRT before sensitivity analysis. **(D)** 5-year DFS rate for RT vs. NRT after sensitivity analysis and excluding the study that brought about heterogeneity. UAC, uterine cervical adenocarcinoma; RT, radiotherapy; NRT, non-radiotherapy; OS, overall survival; DFS, disease-free survival; Surv, survival; OR, odds ratio; 95% CI, 95% confidence interval.

In this study, we firstly conducted a meta-analysis using the available literatures to investigate the effect of RT on the prognosis of UAC patients (26–30). After searching, de-duplicating and screening the literatures, five studies were finally included in the meta-analysis, of which one was RCT and four were case-control studies. Of the five studies, four included UAC patients with FIGO stages IB-IIA, except for the study by Kondo et al. (30) which included a population covering FIGO stages I-IV. RCT and one retrospective study compared survival outcomes in UAC patients undergoing radical RT and radical surgery, whereas three other retrospective studies reported the impact of adjuvant RT on the prognosis of UAC patients (**Supplementary Table S1**). A meta-analysis of the data extracted from all these 5 studies suggested that RT did not have a significant effect on OS in patients with UAC (OR: 0.91, 95% CI 0.30 - 2.75), and significant heterogeneity was noted among the studies (I^2^ = 69.28%, H^2^ = 3.26) (**Figure 1A**). Accordingly, we further performed a sensitivity analysis and found that the heterogeneity originated from the studies by Zhang et al. (26) and Kondo et al. (30) Therefore, we excluded these 2 studies, and the remaining 3 included UAC patients with FIGO stage IB-IIA. Upon analysis, RT wasn’t found to improve OS in this cohort with FIGO stage IB-IIA (OR: 0.44, 95% CI 0.18 - 1.07) and low heterogeneity across studies was demonstrated (I^2^ = 0.73%, H^2^ = 1.01) (**Figure 1B**). Four of the five studies documented 5-year DFS rates in UAC patients. A meta-analysis of these 4 studies revealed that RT did not improve DFS in these patients (OR: 1.11, 95% CI 0.33 - 3.71), however there was significant heterogeneity among studies (I^2^ = 56.27%, H^2^ = 2.29) (**Figure 1C**). Therefore, we performed a sensitivity assessment and after excluding the study by Zhang et al. (26) that brought about heterogeneity, further analyzed and found that RT had no significant effect on DFS in FIGO stage IB-IIA UAC patients (OR: 0.72, 95% CI 0.34 - 1.52; I^2^ = 0.00%, H^2^ = 1.00) (**Figure 1D**).

### 3.2 The effect of RT on CSS from UAC and identify potential participants benefiting from RT

The above analysis initially revealed the impact of RT on OS and DFS in UAC patients with FIGO stage IB-IIA. However, it is worth noting that OS was also confounded by factors other than UAC, and therefore we subsequently analyzed the effect of RT on CSS using data from the SEER database. After screening, 14,649 patients with pathologically confirmed UAC during 2000 – 2019 were included in this study from the SEER database. The median age of these patients was 47 (range 39 - 59) years. The 3-year CSS rate and 5-year CSS rate were 60.9% and 49.1%, respectively, and the median survival time was 58 months. Of these patients, 8,145 patients received RT (RT group) and 6,504 patients did not receive RT (NRT group). Significant differences were observed between the two groups in terms of age, race, pathologic tumor, node, metastasis (pTNM) stage, grade, tumor size, surgery for distant lesions, surgery for primary tumor, lymph node dissection (LND) and ChT. To increase comparability between groups and to equalize confounding factors, we performed PSM in a 1:1 ratio between RT and NRT groups, with 2,191 cases in each group. No statistical significance was found for the differences in pTNM stage, ChT or not, and surgery or not for distant metastatic tumors between the two groups (**Table 2**). The whole cohort consisted of 2,661 cases (60.7%) in pT_1_ stage, 812 cases (18.5%) in pT_2_ stage, 636 cases (14.5%) in pT_3_ stage, 273 cases (6.3%) in pT_4_ stage, 3,339 cases (76.2%) in pN_0_ and 1,043 cases (23.8%) in pN_1_ stage, and 3,476 cases (79.3%) in pM_0_ and 906 cases (20.7%) in pM_1_ stage, respectively, suggesting that all stages were covered rather than only early or advanced stage.

**Table 2:** Baseline characteristics of UAC patients before and after PSM between January 2000 and December 2019 from the SEER database.

	Before PSM			After PSM		
	NRT (N = 8145)	RT (N = 6504)	*P* ^1^	NRT (N = 2191)	RT (N = 2191)	*P* ^1^
Age (years)						
<=49	5243 (64%)	2977 (46%)	<0.001***	800 (37%)	937 (43%)	<0.001***
50-69	2205 (27%)	2612 (40%)		920 (42%)	844 (39%)	
>=70	697 (9%)	915 (14%)		471 (21%)	410 (19%)	
Race						
white	6569 (81%)	5123 (79%)	<0.001***	1676 (76%)	1655 (76%)	0.66
black	540 (7%)	641 (10%)		261 (12%)	263 (12%)	
other	1036 (13%)	740 (11%)		254 (12%)	273 (12%)	
Marital status						
single	1962 (24%)	1612 (25%)	0.641	527 (24%)	577 (26%)	0.709
married or ever married	5558 (68%)	4654 (72%)		1664 (76%)	1614 (74%)	
NA	625 (7.7%)	238 (3.7%)				
Multi-primary tumors						
One primary only	6861 (84%)	5415 (83%)	0.249	1677 (77%)	1707 (78%)	0.040*
1st of 2 or more primaries	620 (8%)	575 (9%)		203 (9.3%)	226 (10%)	
2nd or more of primaries	664 (8%)	514 (8%)		311 (14%)	258 (12%)	
Grade						
grade I	2211 (27%)	870 (13%)	<0.001***	459 (21%)	502 (23%)	0.040*
grade II	2268 (28%)	1873 (29%)		779 (36%)	817 (37%)	
grade III-IV	1391 (17%)	2308 (35%)		953 (43%)	872 (40%)	
NA	2275 (27.9%)	1453 (22.3%)				
Tumor size(mm)						
≤19	3066 (38%)	542 (8%)	<0.001***	521 (24%)	605 (28%)	0.010*
20-39	1318 (16%)	1275 (20%)		722 (33%)	709 (32%)	
>39	769 (9%)	2867 (44%)		948 (43%)	877 (40%)	
NA	2992 (36.7%)	1820 (28.0%)				
pT stage						
T_1_	6679 (82%)	2959 (45%)	<0.001***	1329 (61%)	1332 (61%)	0.87
T_2_	352 (4%)	2070 (32%)		405 (18%)	407 (19%)	
T_3_	295 (4%)	964 (15%)		314 (14%)	322 (15%)	
T_4_	141 (2%)	202 (3%)		143 (6.5%)	130 (5.9%)	
NA	678 (8.3%)	309 (4.8%)				
pN stage						
N_0_	6927 (85%)	4052 (62%)	<0.001***	1670 (76%)	1669 (76%)	0.97
N_1_	478 (6%)	1939 (30%)		521 (24%)	522 (24%)	
NA	740 (9.1%)	513 (7.9%)				
pM stage						
M_0_	7269 (89%)	5516 (85%)	<0.001***	1727 (79%)	1749 (80%)	0.41
M_1_	620 (8%)	906 (14%)		464 (21%)	442 (20%)	
NA	256 (3.1%)	82 (1.3%)				
Surgery for primary site						
no	1207 (15%)	3123 (48%)	<0.001***	824 (38%)	746 (34%)	0.014*
yes	6899 (85%)	3373 (52%)		1367 (62%)	1445 (66%)	
NA	39 (0.5%)	8 (0.1%)				
Surgery for lymph nodes						
no	3027 (37%)	3941 (61%)	<0.001***	1265 (58%)	1158 (53%)	0.001**
yes	5029 (62%)	2516 (39%)		926 (42%)	1033 (47%)	
NA	89 (1.1%)	47 (0.7%)				
Surgery for distant lesions						
no	7594 (93%)	5943 (91%)	<0.001***	2019 (91.1%)	1992 (90.9%)	0.14
yes	498 (6%)	547 (8%)		172 (7.9%)	199 (9.1%)	
NA	53 (0.7%)	14 (0.2%)				
ChT						
no	7501 (92%)	1602 (25%)	<0.001***	1768 (71%)	1573 (69%)	0.086
yes	644 (8%)	4902 (75%)		628 (29%)	680 (31%)	

^1^Wilcoxon rank sum test; Pearson’s Chi-squared test.

*, two-sided P values < 0.05, **, two-sided P values < 0.01, ***, two-sided P values < 0.001. UAC, uterine cervical adenocarcinoma; PSM, propensity score matching; NA, unknown data; pT stage, pathologic stage of primary tumor; pN stage, pathologic stage of lymph nodes; pM stage, pathologic stage of metastasis, RT, radiotherapy; NRT, non-radiotherapy; SEER, surveillance, epidemiology and end results; ChT, chemotherapy.

**Figure 2:**
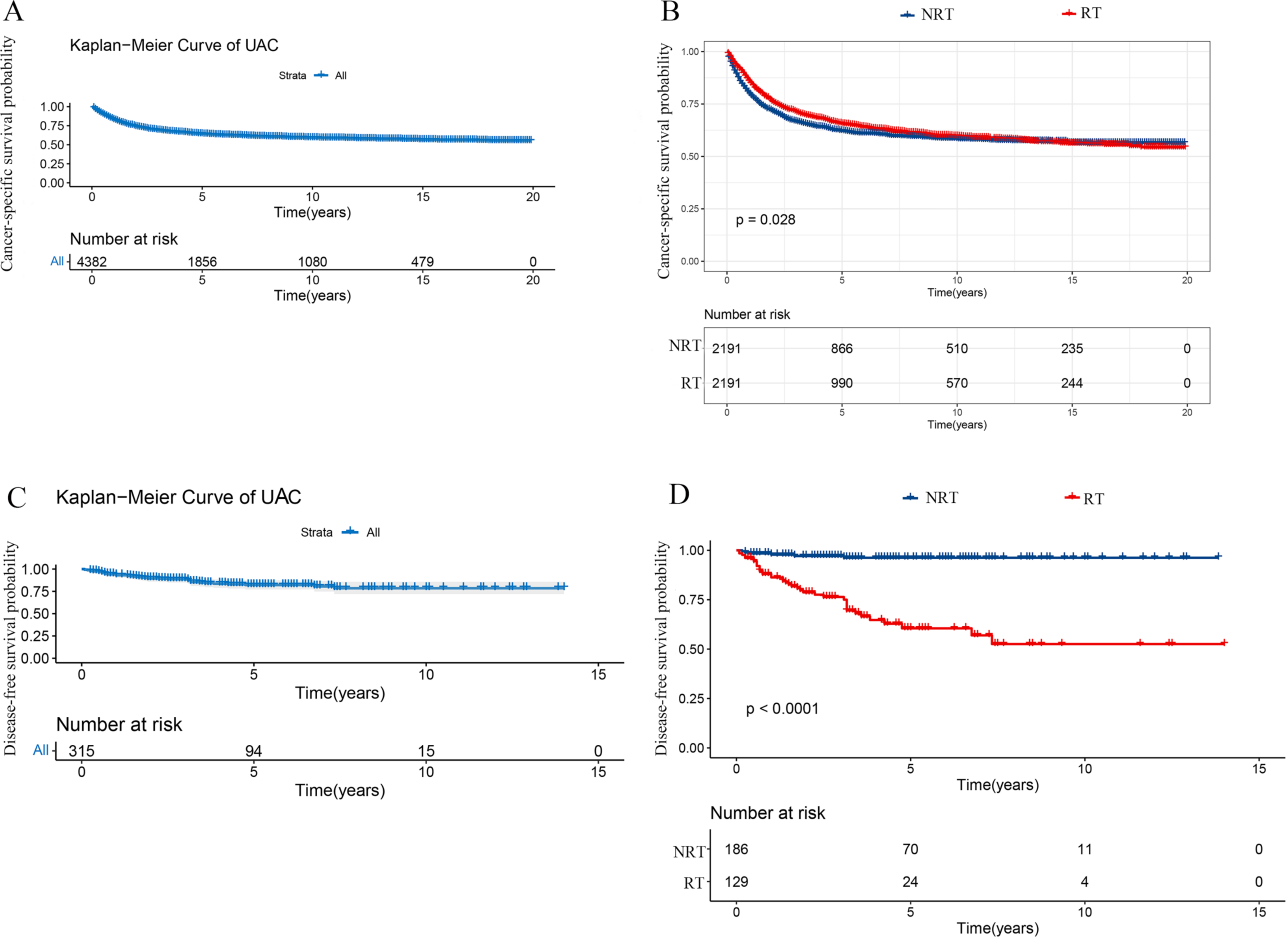
Kaplan-Meier curves in patients with UAC undergoing RT or NRT. **(A)** Kaplan-Meier curve of CSS in 4382 patients with UAC from the SEER database. **(B)** Superior CSS was found in the RT group to the NRT group (P = 0.028). **(C)** Kaplan-Meier curve of DFS in 315 patients with UAC from our center. **(D)** Poorer DFS was observed in the RT group than the NRT group (P < 0.001). UAC, uterine cervical adenocarcinoma; RT, radiotherapy; NRT, non-radiotherapy; SEER, surveillance, epidemiology and end results; CSS, cancer specific survival; DFS, disease-free survival.

**Figure 3:**
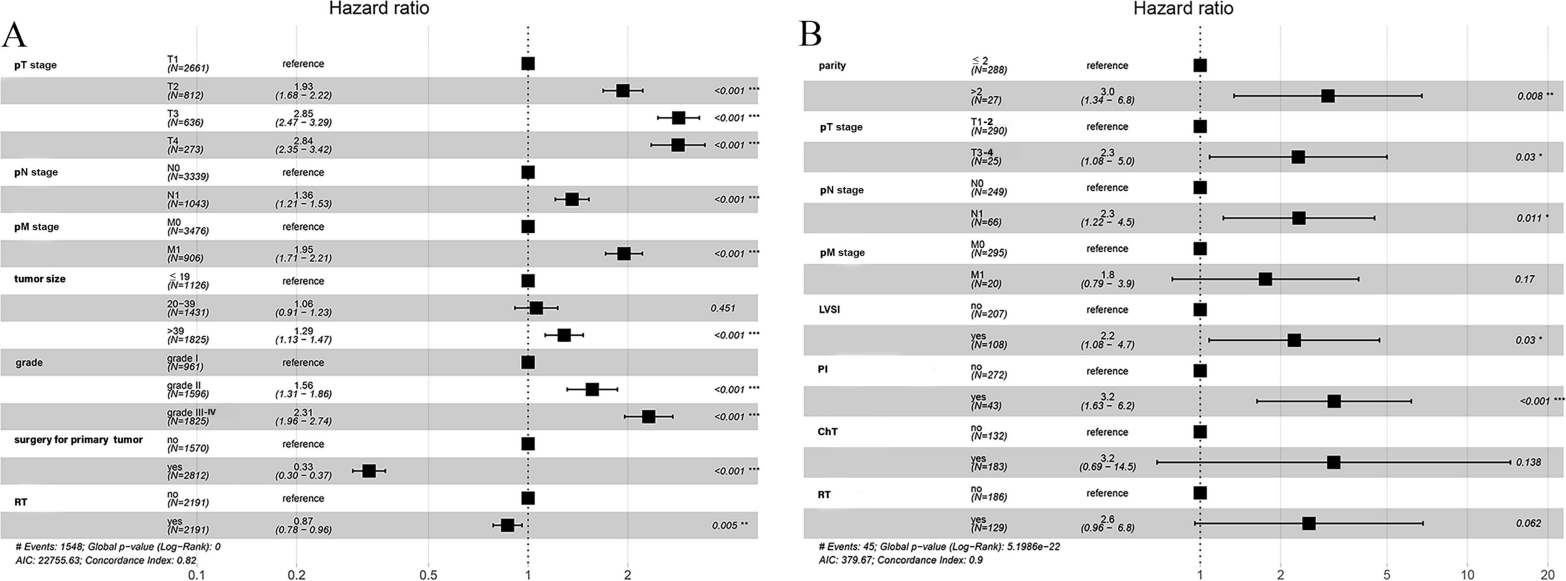
Forest plot demonstrating independent factors for predicting CSS and DFS in UAC patients with multivariate Cox regression analysis. **(A)** Factors for predicting CSS in UAC patients from the SEER database. **(B)** Factors for predicting DFS in UAC patients from our center. *, two-sided P values < 0.05; **, two-sided P values < 0.01; ***, two-sided P values < 0.001; UAC, uterine cervical adenocarcinoma; CSS, cancer specific survival; DFS, disease-free survival; SEER, surveillance, epidemiology and end results; pT stage, pathologic stage of primary tumor; pN stage, pathologic stage of lymph nodes; pM stage, pathologic stage of metastasis; LVSI, lymph-vascular space invasion; RT, radiotherapy; ChT, chemotherapy.

Upon analysis of the matched population, the 3- and 5-year CSS rates were 69.0% and 63.9%, respectively (**Figure 2A**). Compared with the NRT group, CSS was superior in the RT group (HR: 0.90, 95% CI 0.81 - 0.99, P = 0.028), with 3-year CSS rates and 5-year CSS rates of 72.4% and 63.8%, respectively, while 67.1% and 62.5 in the NRT group, respectively (**Figure 2B**). In this study, Cox proportional hazard model was used to analyze the independent predictors affecting CSS in patients with UAC. We initially tested the model for proportionality of risk assumptions and analyzed multicollinearity across all variables and found multicollinearity between LND and pN stage with a variance inflation factor (VIF) of 6.2, and therefore the variable LND no longer included in subsequent analyses. In addition, to prevent overfitting of the model, LASSO regression analysis was employed in this study to screen the variables. Subsequently, following univariate (**Supplementary Table S2**) and multivariate Cox regression analyses, we found that UAC patients who underwent RT (HR: 0.87, 95% CI 0.78 - 0.96, P = 0.005) and surgery for primary tumor (HR: 0.33, 95% CI 0.30 - 0.37, P < 0. 001) had a better CSS while patients with stage pT2-4, pN1, pM1, tumors > 39 mm, and histological grade ≥ II presented worse CSS, and a forest plot displayed the result (**Figure 3A**).

**Figure 4:**
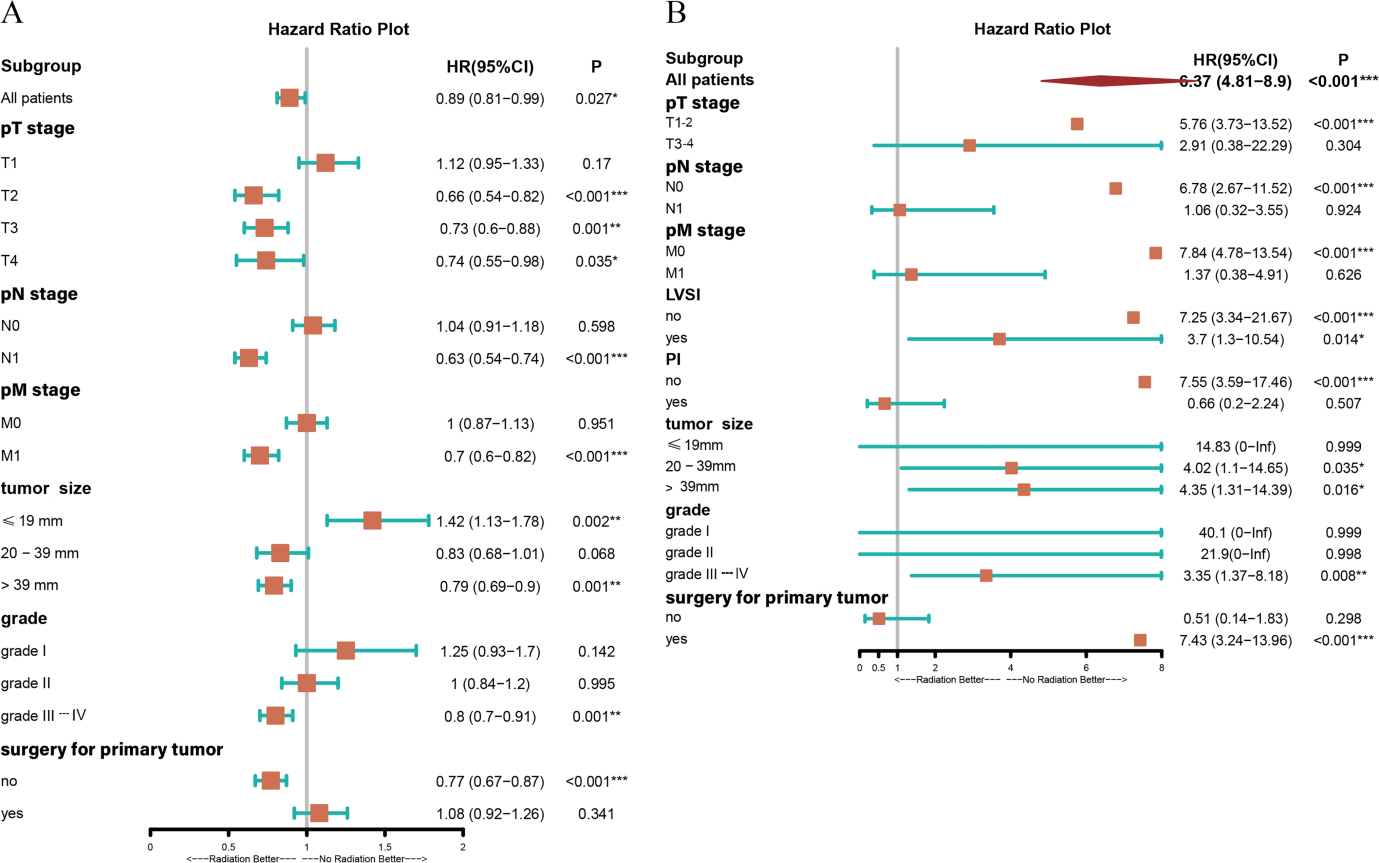
Forest plot demonstrating the subgroup analysis for patients with UAC in the presence of RT or NRT based on different conditions. **(A)** Subgroup analysis of CSS for patients with UAC from the SEER database in the presence of RT or NRT based on different conditions. **(B)** Subgroup analysis of DFS for patients with UAC from our center in the presence of RT or NRT based on different conditions. *, two-sided P values < 0.05; **, two-sided P values < 0.01; ***, two-sided P values < 0.001; UAC, uterine cervical adenocarcinoma; RT, radiotherapy; NRT, non-radiotherapy; SEER, surveillance, epidemiology and end results; CSS, cancer specific survival; DFS, disease-free survival; pT stage, pathologic stage of primary tumor; pN stage, pathologic stage of lymph nodes; pM stage, pathologic stage of metastasis; LVSI, lymph-vascular space invasion; PI, parametrial involvement; HR, hazard ratio; CI, confidence interval.

**Figure 5:**
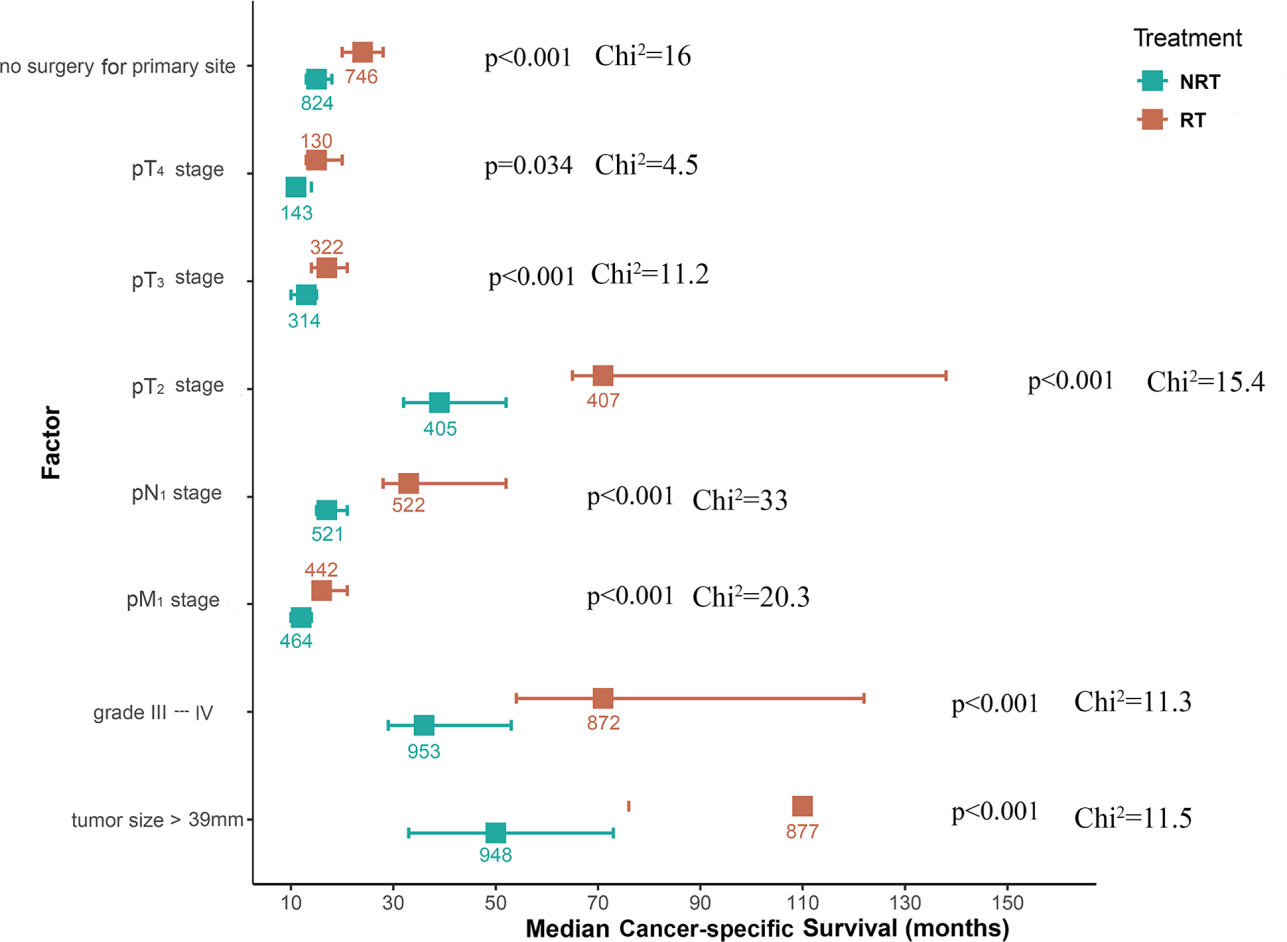
Forest plot of median CSS time across different situations for UAC patients from the SEER database treated with RT or NRT during subgroup analyses. UAC, uterine cervical adenocarcinoma; RT, radiotherapy; NRT, non-radiotherapy; CSS, cancer specific survival; pT stage, pathologic stage of primary tumor; pN stage, pathologic stage of lymph nodes; pM stage, pathologic stage of metastasis; SEER, surveillance, epidemiology and end results.

The aforementioned results confirmed that RT improved CSS in patients with UAC. To explore the possibility that all UAC patients may benefit from RT, a subgroup analysis was conducted. It was found that UAC patients with primary tumors beyond the cervix, or with lymph node metastasis, or with distant metastasis, or with primary tumors larger than 39 mm in diameter, or with histological grade III-IV, or with unresected primary tumors who were treated with RT achieved better CSS (**Figure 4A**) and longer median CSS time (**Figure 5**) compared with those who did not receive RT. And when the tumor stage was pT_1_, or pN_0_, or pM_0_, receiving RT or not did not affect these patients’ CSS (**Figure 4A**). Instead, patients with primary tumor diameters ≤ 19 mm who received RT had an increased risk of UAC specific death (**Figure 4A**), and this population had not yet reached the median CSS time, so it is not shown in **Figure 5**.

### 3.3 The effect of RT on DFS from UAC

According to the guidelines, besides factors like TNM staging, tumor size and grading, LVSI, depth of stromal invasion, PI, and vaginal margin status are also important factors affecting the prognosis of UAC (31). Nonetheless, the SEER database lacks information on the above variables as well as tumor recurrence. Accordingly, we further included 315 patients with pathologically confirmed UAC at our center from January 2010 to December 2023 to explore the effect of RT on their DFS. The median age of these patients was 46 years (range 39 - 53) and there were 218 (69.3%), 27 (8.5%), 45 (14.2%) and 25 (7.9%) FIGO 2018 Stage I, II, III and IV cases, respectively. Of these patients, 283 (89.8%) underwent primary tumor resection and 32 (10.2%) did not. 129 (41.0%) cases were given RT (RT group) and 186 (59.0%) did not receive RT (NRT group). (**Table 3**).

**Table 3:** Baseline characteristics of patients diagnosed with UAC between January 2010 and December 2023 at our center.

	NRT (N = 186)	RT (N = 129)	*P* ^1^
Age (years)			
≤ 49	124 (66.7%)	65 (50.4%)	0.005**
≥ 50	62 (33.3%)	64 (49.6%)	
Marital status			
single	12 (6.5%)	5 (3.9%)	0.459
married or ever married	174 (93.5%)	124 (96.1%)	
Gravidity			
≤ 3	134 (72.0%)	92 (71.3%)	0.989
> 3	52 (28.0%)	37 (28.7%)	
Parity			
≤ 2	170 (91.4%)	118 (91.5%)	1
> 2	16 (8.6%)	11 (8.5%)	
FIGO stage			
Stage I and II	178 (95.7%)	67 (51.9%)	<0.001***
Stage III and IV	8 (4.3%)	62 (48.1%)	
pT stage			
pT_1-2_	182 (97.8%)	108 (83.7%)	<0.001***
pT_3_-_4_	4 (2.2%)	21 (16.3%)	
pN stage			
pN_0_	177 (95.2%)	72 (55.8%)	<0.001***
pN_1_	9 (4.8%)	57 (44.2%)	
pM stage			
pM_0_	181 (97.3%)	114 (88.4%)	0.003***
pM_1_	5 (2.7%)	15 (11.6%)	
Tumor size (mm)			
≤ 19	109 (58.6%)	11 (8.5%)	<0.001***
20-39	49 (26.3%)	50 (38.8%)	
>39	28 (15.1%)	68 (52.7%)	
Grade			
grade I	81 (43.5%)	20 (15.5%)	<0.001***
grade II	63 (33.9%)	49 (38.0%)	
grade III-IV	42 (22.6%)	60 (46.5%)	
Histology			
usual type	148 (79.6%)	85 (65.9%)	0.01**
unusual type	38 (20.4%)	44 (34.1%)	
HPV status			
negative	22 (11.8%)	35 (27.1%)	<0.001***
positive	131 (70.3%)	64 (49.6%)	
NA	33(10.5%)	30(9.5%)	
Corpus involvement			
no	156 (83.9%)	62 (48.1%)	<0.001***
yes	30 (16.1%)	67 (51.9%)	
Depth of invasion			
superficial 1/3	129 (69.4%)	15 (11.6%)	<0.001***
middle 1/3	33 (17.7%)	31 (24.0%)	
deep 1/3	24 (12.9%)	83 (64.3%)	
LVSI			
no	153 (82.3%)	54 (41.9%)	<0.001***
yes	33 (17.7%)	75 (58.1%)	
Parametrial involvement			
no	181 (97.3%)	91 (70.5%)	<0.001***
yes	5 (2.7%)	38 (29.5%)	
Margin status			
negative	179 (96.2%)	95 (73.6%)	0.106
positive	3 (1.6%)	6 (4.7%)	
missing	4 (2.2%)	28 (21.7%)	
Surgery for primary site			
no	4 (2.2%)	28 (21.7%)	<0.001***
yes	182 (97.8%)	101 (78.3%)	
Lymph nodes dissection			
no	37 (19.9%)	26 (20.2%)	1
yes	149 (80.1%)	103 (79.8%)	
ChT			
no	119 (64.0%)	13 (10.1%)	<0.001***
yes	67 (36.0%)	116 (89.9%)	

^1^Wilcoxon rank sum test; Pearson’s Chi-squared test.

*, two-sided P values < 0.05, **, two-sided P values < 0.01, ***, two-sided P values < 0.001. UAC, uterine cervical adenocarcinoma; FIGO, International Federation of Gynecology and Obstetrics; pT stage, pathological stage of primary tumor; pN stage, pathological stage of lymph nodes; pM stage, pathological stage of metastasis; RT, radiotherapy; NRT, non-radiotherapy; HPV, human papilloma virus; NA, unknown data; LVSI, lymph-vascular space invasion; ChT, chemotherapy.

After a median follow-up of 40 (range 19 - 67) months, a total of 45 deaths were reported, with 3- and 5-year DFS rates of 87.5% and 82.1%, respectively. (**Figure 2C**). After analyzing the effect of RT on DFS with Kaplan-Meier method, it was found that the RT group in our center exhibited inferior DFS to the NRT group (P < 0.001) (**Figure 2D**). To investigate the independent prognostic factors affecting DFS in patients with UAC, we included factors other than the aforementioned variables extracted from the SEER database, such as LVSI, depth of stromal invasion, PI, and vaginal margin status, and analyzed them using the Cox proportional hazard model. After univariate Cox regression analysis (**Supplementary Table S3**), we found that the P values of all 18 factors were less than 0.05, except for marital status, number of gravidities, and histologic type. To prevent overfitting of the model, LASSO regression was as well employed in this part of the study, and ultimately 8 variables were selected to enter the subsequent analysis, including: number of parity, pTNM stage, LVSI, PI, ChT, and RT. Multivariate Cox regression analysis revealed that pM stage, ChT, and RT were not independent prognostic factors affecting DFS, whereas number of parity > 2 (HR: 3.0, 95% CI 1.3 - 6.8), pT3-4 (HR: 2.3, 95% CI 1.1 - 5.0), pN1 stage (HR: 2.3, 95% CI 1.2 - 4.5), the presence of LVSI (HR: 2.2, 95% CI 1.1 - 4.7) and PI (HR: 3.2, 95% CI 1.6 - 6.2) increased the risk of recurrence in patients with UAC, which were shown in a forest plot (**Figure 3B**).

To explore which patients could have potential DFS benefit from RT, we also performed subgroup analyses and observed that patients with PI as well as those without resection for primary tumor showed a trend to benefit from RT, but with a P value > 0.05. However, when the tumor had a stage of pT_1-2_, pN_0_, pM_0_, no PI, size of > 19 mm, grades III-IV, with or without LVSI, and with resection for primary tumor, patients treated with RT showed inferior DFS to those who did not receive RT (**Figure 4B**). It is worth noting that due to the limited number of UAC patients in our center, there were less than 10 cases with stage pT_3-4_, pN_1_, pM_1_, and the presence of PI, respectively, who also did not undergo RT. Therefore, the effect of RT on DFS in this population needs to be further confirmed by expanding the sample size.

## 4 Discussion

We evaluated the effect of RT on OS, CSS, and DFS in UAC patients based on meta-analyses and retrospective analyses of different populations. It was found that RT improved CSS in patients with UAC, but the effect on OS and DFS was not statistically significant. Those with stage pT_2-4_, pN_1_, pM_1_, primary tumor size greater than 39 mm, tumor grade III-IV, or unresected primary tumors had superior CSS when treated with RT than those who did not receive RT. In terms of DFS, RT is not an independent prognostic factor. For cases staged as pT_1-2_, pN_0_, pM_0_, with tumors larger than 19 mm, graded III-IV, with primary tumors resected, without PI, and either with or without LVSI, the DFS of individuals who received RT was inferior to those who did not receive RT.

In clinical practice, UAC is mostly considered to be less sensitive to RT. Accordingly, even in locally advanced UAC patients, such as with FIGO 2018 stage IB3, IIA2, and selected stage IIB, physicians tend to prefer surgical treatment over CCRT (31). Nevertheless, there are few studies and inconsistent results as to whether RT can provide advantages for patients with UAC and which populations would benefit from RT, as confirmed by the literatures included in the meta-analysis section of this study. We analyzed the impact of RT on CSS in patients with UAC using data from the SEER database between 2000 and 2019. PSM was introduced in this study for balancing the differences in important factors like pTNM staging and receipt of ChT or not between the RT group and the NRT group. The results revealed that the CSS of RT group was more favorable than that of NRT group, and RT was identified as an independent factor affecting the CSS of UAC patients upon multivariate Cox regression analyses, as well as pTNM staging, surgery for the primary tumor, tumor size and histological grading. We further explored the population that might benefit from RT by subgroup analysis and found that those patients staged at pT_2-4_, pN_1_, and pM_1_, with tumor diameter ≥ 39 mm, histological grades III-IV, and who had not received surgery for the primary tumor experienced a superior CSS with RT compared to those who did not receive RT. Among the populations, those with more advanced stages and unresected primary tumors benefited from RT, which is consistent with the findings of Wang et al. (32). A retrospective analysis by Zhou et al. (33). also found that a superior CSS was noted in those patients with lymph node metastases who underwent surgery combined with postoperative aRT compared to surgery alone. In contrast, Fa et al. (34). indicated that OS in patients with UAC was not related to RT, but rather to age, marital status, tumor size, histological grade, FIGO stage, pelvic lymph node metastasis, surgery, and ChT. An increased risk of death was observed in patients with UAC who received RT compared to those who did not (HR: 1.39, 95% CI 1.12 - 1.72) in a study by Chen et al. (35). As seen, previous studies have shown inconsistent results in terms of the effect of RT on survival outcomes in patients with UAC, but our study confirmed, utilizing a PSM approach, that RT can provide a CSS benefit to them. Of note, UAC covers multiple histologic subtypes with inconsistent sensitivity to RT, and the inclusion of different subtypes in different studies can lead to bias.

The above findings demonstrated the impact of RT on CSS in patients with UAC, and it is of concern whether RT can similarly affect the recurrence of UAC. Due to the lack of information on tumor recurrence in the SEER database, this portion of the study included the UAC cohort from our center for analysis. As a result, RT was not identified as an independent prognostic factor for DFS in UAC patients, whereas parity > 2, pT_3-4_, pN_1_, presence of LVSI and PI were independent risk factors. Interestingly, the increased risk of recurrence found here in patients with parity > 2 is considered to be related to cervical or vaginal injury due to multiple births, which has not been reported in the available literatures. Of note, the population included in this part of the study was from the gynecology department of a general hospital, a surgical-oriented department, where most of the patients were at early stages, with 245 (77.8%) FIGO stage I-II patients and 45 (14.3%) FIGO stage III patients, of which 43 (95.6%) FIGO stage III patients had a primary tumor of pT1–2 with lymph node metastasis. Therefore, the majority of patients (283/315) underwent radical surgery, which may have potentially contributed to the bias. The seemingly paradoxical effects of radiotherapy on CSS and DFS were not in conflict, which was confirmed by the further subgroup analyses. It was found that compared with NRT, those with tumors of pT_1-2_, pN_0_, pM_0_, no PI, and those with primary tumors resection underwent RT presented inferior DFS, which is consistent with previous reports. A study by Ouyang et al. (36) confirmed that UAC patients with pT1-2aN0M0 who received adjuvant RT after radical surgery experienced an increased risk of death (HR: 1.78, 95% CI 1.26 - 2.51, P < 0.001). Chen et al. (37) included 258 patients with stage IB1-IIA UAC to explore the prognostic impact of different treatment modalities, and found that those with 1 risk factor who underwent RT after surgery had a 2.8-fold increased risk of disease recurrence (P = 0.001) and a 3.2-fold increased risk of disease-related death (P < 0.001), compared with patients in the surgery-only group. The findings suggest that RT does not enhance DFS in UAC patients at low risk. Conversely, the RT-related toxicity, involving lower gastrointestinal tract, bladder and vagina, and their impact on quality of life may potentially impair the survival outcomes. Our study also observed that RT did not improve DFS in patients with UAC regardless of the presence of LVSI (37), which seems to be in conflict with the recommendations of the four-factor model of UAC (38), and this finding may be due to the influence of ChT as well as the limited sample size. All the UAC patients with LVSI in our center were treated with ChT. Due to the limited number of UAC patients in our center, less than 10 cases were not treated with RT among those with stage pT_3-4_, pN_1_, pM_1_ and presence of PI, respectively. Consequently, this result can only indicate that RT failed to improve CSS in patients with pT_1-2_, with no lymph node metastasis and with no distant metastasis. The influence of RT on DFS in the population with pT_3-4_, pN_1_ and pM_1_ needs to be further investigated by expanding the sample size.

The present study comprehensively analyzed the effect of RT on the prognosis of patients with UAC through a combination of meta-analysis and retrospective study by using populations from different sources, by employing PSM method to reduce intergroup differences, by multivariate cox regression analyses to control for covariates, and by focusing on OS, CSS, and DFS. OS considers all causes of death, which are primarily determined by the aggressiveness of the tumor and various treatments, but are also influenced by comorbidities and treatment-related complications, whereas CSS only assesses the impact of a specific cause, as UAC, on survival. DFS time is primarily determined by the interval between tumor recurrences, but it is also influenced by the frequency of follow-up, the presence of other comorbidities and adverse effects. To strictly evaluate the effect of RT on UAC-specific survival rates and tumor recurrence, we separately employed CSS and DFS as the primary outcome in the following studies. We also explored populations that may potentially benefit from RT using subgroup analyses. The meta-analysis portion was performed in rigorous accordance with PRISMA standards with a high level of evidence. The present study entails the following limitations. First, due to the low prevalence of UAC, the literatures included in the meta-analysis part of this study were retrospective, with the exception of one prospective RCT study. Second, the analysis of the effect of RT on CSS and DFS in patients with UAC was based on a retrospective study and the small number of FIGO stage III-IV patients at our center, with only 4 cases in this population not receiving RT. Third, the available information in the SEER database spans nearly 20 years, and the development of RT techniques and the evolution of ChT regimens may have interfered with the results. Fourth, due to the lack of HPV status in the SEER database and the limited number of UAC patients in our center, stratified analyses based on HPV status and different histological subtypes, i.e., ordinary adenocarcinoma, clear cell carcinoma, and mucinous adenocarcinoma, were not performed. In addition, with the progress of UAC-related studies, especially the application of Silva typing and the improvement of RT techniques, and different histological subtypes with varying sensitivity to RT, further prospective RCTs with enlarged sample sizes and inclusion of more variables are warranted in the future.

## 5 Conclusion

This study established that RT improved CSS but not OS and DFS in patients with UAC. A superior CSS was obtained in patients who underwent RT compared to those who did not, when they have a primary tumor beyond the cervix, or lymph node metastasis, or distant metastasis, or tumor size > 39 mm, or histologic grade III-IV, or an unresected primary tumor. Nonetheless, patients with pT_1-2_, N_0_ or M_0_, no PI, tumor size greater than 19 mm, histological grade III-IV, resection of the primary tumor, and with or without LVSI treated with RT presented a worse DFS. These findings provide evidence for the decision of the optimal treatment modality for patients with UAC and also for future prospective studies.

## Data Availability

Publicly available datasets were analyzed in this study. This data can be found here: http://seer.cancer.gov/seerstat.

## Supplementary material

Supplementary Figure 1 Flowchart of data processing for this study. **(A)** Literature screening process for the systematic review and meta-analysis. **(B)** Flowchart of data processing and analysis from the SEER database. **(C)** Flowchart of data processing and analysis from our center. CC, cervical cancer; UAC, uterine cervical adenocarcinoma; WHO, World Health Organization, IECC, International Endocervical Criteria and Classification; SEER, surveillance, epidemiology and end results; CSS, cancer specific survival; DFS, disease-free survival; AJCC, American Joint Committee on Cancer; RT, radiotherapy; NRT, non-radiotherapy.
