# Supplementary figures and images for: Impact of radiotherapy on the prognosis in uterine cervical adenocarcinoma: a meta-analysis and retrospective cohort study
Source: Front Oncol. 2025 Sep 9;15:1653107. doi: 10.3389/fonc.2025.1653107 (PMC12455619; doi:10.3389/fonc.2025.1653107)

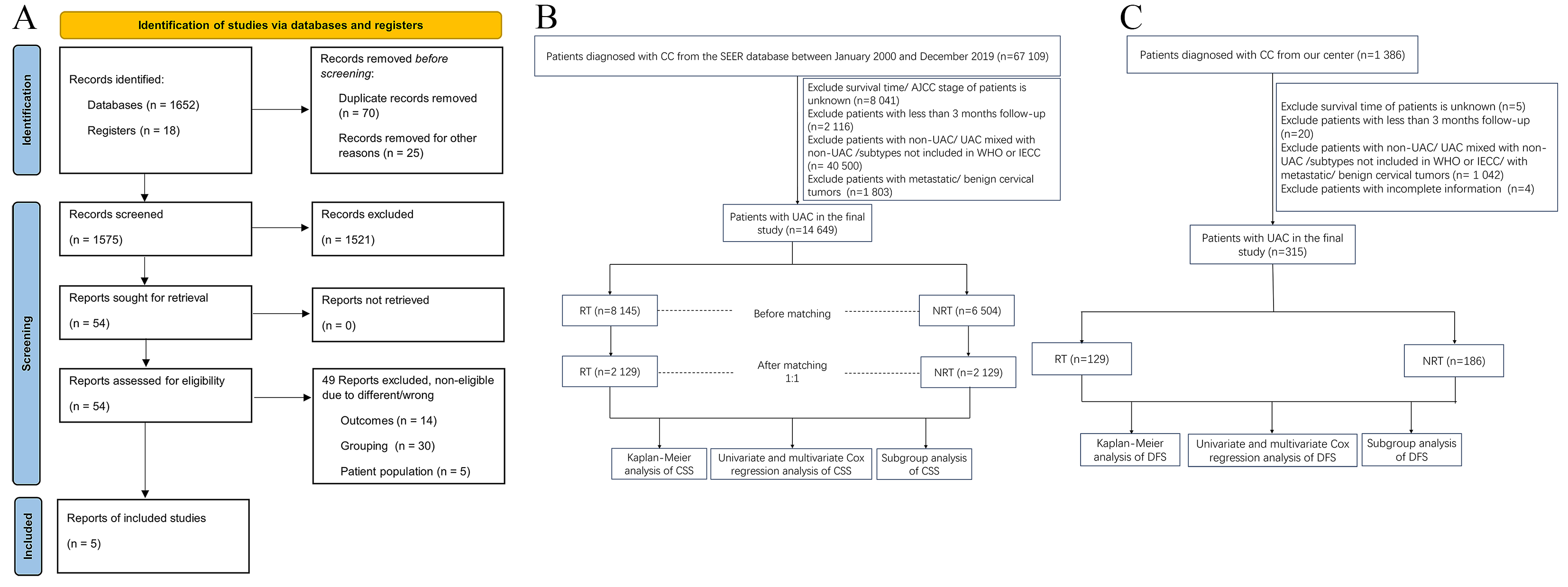

Supplement: Supplementary Figure 1 — Flowchart of data processing for this study. (A) Literature screening process for the systematic review and meta-analysis. (B) Flowchart of data processing and analysis from the SEER database. (C) Flowchart of data processing and analysis from our center. CC, cervical cancer; UAC, uterine cervical adenocarcinoma; WHO, World Health Organization, IECC, International Endocervical Criteria and Classification; SEER, surveillance, epidemiology and end results; CSS, cancer specific survival; DFS, disease-free survival; AJCC, American Joint Committee on Cancer; RT, radiotherapy; NRT, non-radiotherapy. [file Image1.tif]
