# Supplementary material for: Impact of radiotherapy on the prognosis in uterine cervical adenocarcinoma: a meta-analysis and retrospective cohort study
Source: Front Oncol. 2025 Sep 9;15:1653107. doi: 10.3389/fonc.2025.1653107 (PMC12455619; doi:10.3389/fonc.2025.1653107)
Supplement: Supplementary file 2 [file Table1.docx]

Supplementary Material

**Supplementary Tables**

**Table S1: Characteristics of the studies included in the systematic review and meta-analysis.**

^1^

| Author | Published year | Country | Study design | Tumor stage | Intervention (n) | Radiation methods (dose) | |  | Outcome measures |
| --- | --- | --- | --- | --- | --- | --- | --- | --- | --- |
| Landoni, F.et al | 1997 | Italy | randomized | IB and IIA^1^ | RS (26) vs EBRT+BRT (20) | BRT (NA) and EBRT (47 Gy) | | | 5-year OS; 5-year DFS |
| Ouyang, P.et al | 2020 | China | retrospective | IB2 and IIA2^2^ | RS+NACT (24) vs RS+NACRT (38) | BRT (12 Gy) and EBRT (45 Gy) | | | 5-year OS; 5-year DFS |
| Feng, Z.et al | 2017 | China | retrospective | IB2 and IIA^3^ | RS+ChT (4) vs RS+EBRT+BRT+ChT (31) | BRT (10 Gy) and EBRT (45-50 Gy) | | | 5-year OS; 5-year DFS |
| Kondo, E. et al | 2022 | Japan | retrospective | I-IV^4^ | RS (307) vs RT (307) | NA |  | | 5-year OS |
| Xiaoyi, W, et al | 2017 | China | retrospective | IB-IIA^2^ | RS+ChT (2) vs RS+CRT (34) | EBRT (40-50 Gy) | | | 5-year OS; 5-year DFS |

^1^AJCC 4th edition; ^2^ FIGO 2009; ^3^ FIGO 2002; ^4^ FIGO 1994.

Abbreviations: RS, radical surgery; EBRT: external beam radiotherapy; BRT: brachytherapy; NACT: neoadjuvant chemotherapy; NACRT: neoadjuvant chemoradiotherapy; ChT: chemotherapy; RT: radiotherapy; CRT: chemoradiotherapy; NA, unknown; OS, overall survival rates; DFS, disease-free survival rates; AJCC: American Joint Committee on Cancer staging; FIGO: International Federation of Gynecology and Obstetrics staging criteria; NA, unknown data.
