# Supplementary material for: Impact of radiotherapy on the prognosis in uterine cervical adenocarcinoma: a meta-analysis and retrospective cohort study
Source: Front Oncol. 2025 Sep 9;15:1653107. doi: 10.3389/fonc.2025.1653107 (PMC12455619; doi:10.3389/fonc.2025.1653107)
Supplement: Supplementary file 3 [file Table2.docx]

Supplementary Material

# Supplementary Tables

**Table S2: Univariate Cox regression analysis for predictors of CSS in the 4382 patients with UAC after PSM from the SEER database.**

| Characteristics | Univariate | |
| --- | --- | --- |
|  | HR (95%CI) | *P* |
| Age (years) |  |  |
| ≤ 49 | 1.00 (Reference) |  |
| 50-69 | 1.84 (1.63 ~ 2.07) | <.001*** |
| ≥ 70 | 2.95 (2.58 ~ 3.38) | <.001*** |
| Race |  |  |
| black | 1.00 (Reference) |  |
| other | 0.68 (0.56 ~ 0.82) | <.001*** |
| white | 0.61 (0.53 ~ 0.70) | <.001*** |
| Marital status |  |  |
| married or ever married | 1.00 (Reference) |  |
| single | 1.04 (0.93 ~ 1.17) | 0.508 |
| Multi-primary tumors |  |  |
| one primary only | 1.00 (Reference) |  |
| 1st of 2 or more primaries | 0.55 (0.45 ~ 0.67) | <.001*** |
| Grade |  |  |
| High differentiated | 1.00 (Reference) |  |
| Moderately differentiated | 1.83 (1.54 ~ 2.18) | <.001*** |
| Low differentiated | 3.68 (3.13 ~ 4.33) | <.001*** |
| Tumor size (mm) |  |  |
| ≤ 19 | 1.00 (Reference) |  |
| 20-39 | 1.04 (0.90 ~ 1.20) | 0.621 |
| > 39 | 2.08 (1.83 ~ 2.37) | <.001*** |
| pT stage |  |  |
| pT_1_ | 1.00 (Reference) |  |
| pT_2_ | 3.03 (2.65 ~ 3.46) | <.001*** |
| pT_3_ | 6.86 (6.04 ~ 7.79) | <.001*** |
| pT_4_ | 7.62 (6.44 ~ 9.02) | <.001*** |
| pN stage |  |  |
| pN_0_ | 1.00 (Reference) |  |
| pN_1_ | 3.09 (2.79 ~ 3.42) | <.001*** |
| pM stage |  |  |
| pM_0_ | 1.00 (Reference) |  |
| pM_1_ | 5.01 (4.52 ~ 5.56) | <.001*** |
| Surgery for primary tumor |  |  |
| no | 1.00 (Reference) |  |
| yes | 0.20 (0.18 ~ 0.22) | <.001*** |
| Lymph nodes dissection |  |  |
| no | 1.00 (Reference) |  |
| yes | 0.31 (0.28 ~ 0.35) | <.001*** |
| Surgery for distant lesions |  |  |
| no | 1.00 (Reference) |  |
| yes | 1.34 (1.14 ~ 1.57) | <.001*** |
| RT |  |  |
| no | 1.00 (Reference) |  |
| yes | 0.90 (0.81 ~ 0.99) | 0.028* |
| ChT |  |  |
| no | 1.00 (Reference) |  |
| yes | 2.07 (1.87 ~ 2.30) | <.001*** |

Notes: *, two-sided P values < 0.05, **, two-sided P values < 0.01, ***, two-sided P values < 0.001. Abbreviations: CSS, cancer-specific survival; UAC, uterine cervical adenocarcinoma; PSM, propensity score matching; SEER, surveillance, epidemiology and end results; pT stage, pathological stage of primary tumor; pN stage, pathological stage of lymph nodes; pM stage, pathological stage of metastasis; RT, radiation; ChT, chemotherapy; HR, hazard ratio; CI, confidence interval.
