# Supplementary material for: Impact of radiotherapy on the prognosis in uterine cervical adenocarcinoma: a meta-analysis and retrospective cohort study
Source: Front Oncol. 2025 Sep 9;15:1653107. doi: 10.3389/fonc.2025.1653107 (PMC12455619; doi:10.3389/fonc.2025.1653107)
Supplement: Supplementary file 4 [file Table3.docx]

Supplementary Material

# Supplementary Tables

**Table S3: Univariate Cox regression analysis for predictors of DFS in the 315 patients with UAC from our center.**

| Characteristics | DFS | |
| --- | --- | --- |
|  | HR (95%CI) | *P* |
| Age (years) |  |  |
| ≤49 | 1.00 (Reference) | 0.049* |
| >49 | 1.80 (1.01 ~ 3.25) |  |
| Marital status |  |  |
| single | 1.00 (Reference) | 0.298 |
| married or ever married | 2.87 (0.39 ~ 20.83) |  |
| Gravidity |  |  |
| ≤3 | 1.00 (Reference) | 0.054 |
| >3 | 1.80 (0.99 ~ 3.27) |  |
| Parity |  |  |
| ≤2 | 1.00 (Reference) | 0.017* |
| >2 | 2.53 (1.18 ~ 5.44) |  |
| FIGO stage |  |  |
| I and II | 1.00 (Reference) | <.001*** |
| III and IV | 21.8 (10.40 ~ 45.60) |  |
| pT stage |  |  |
| pT_1-2_ | 1.00 (Reference) | <.001*** |
| pT_3_-_4_ | 9.31 (4.96 ~ 17.50) |  |
| pN stage |  |  |
| pN_0_ | 1.00 (Reference) | <.001*** |
| pN_1_ | 8.22 (4.48 ~ 15.09) |  |
| pM stage |  |  |
| pM_0_ | 1.00 (Reference) | <.001*** |
| pM_1_ | 14.80 (8.06 ~ 27.14) |  |
| Tumor size (mm) |  |  |
| ≤19 | 1.00 (Reference) | <.001*** |
| 20-39 | 10.4 (2.37 ~ 45.90) |  |
| >39 | 28.2 (6.73 ~ 118.01) |  |
| Grade |  |  |
| grade I | 1.00 (Reference) | <.001*** |
| grade II | 1.08 (0.38 ~ 3.12) |  |
| grade III-IV | 6.16 (2.57 ~ 14.80) |  |
| Histology |  |  |
| usual type | 1.00 (Reference) | 0.280 |
| unusual type | 1.43 (0.75 ~ 2.73) |  |
| Corpus involvement |  |  |
| no | 1.00 (Reference) | <.001*** |
| yes | 7.09 (3.66 ~ 13.72) |  |
| Depth of invasion |  |  |
| superficial 1/3 | 1.00 (Reference) | 0.021* |
| middle 1/3 | 4.94 (1.28 ~ 19.11) | <.001*** |
| deep 1/3 | 20.17 (6.19 ~ 65.66) |  |
| LVSI |  |  |
| no | 1.00 (Reference) | <.001*** |
| yes | 7.23 (3.72 ~ 14.04) |  |
| Parametrial involvement |  |  |
| no | 1.00 (Reference) | <.001*** |
| yes | 13.01 (7.17 ~ 23.63) |  |
| Margin status |  |  |
| no | 1.00 (Reference) | <.001*** |
| yes | 9.47 (3.19 ~ 28.11) |  |
| Surgery for primary tumor |  |  |
| no | 1.00 (Reference) | <.001*** |
| yes | 0.12 (0.06 ~ 0.21) |  |
| Lymph nodes dissection |  |  |
| no | 1.00 (Reference) | 0.007** |
| yes | 0.42 (0.22 ~ 0.79) |  |
| ChT |  |  |
| no | 1.00 (Reference) | <.001*** |
| yes | 17.58 (4.26 ~ 72.60) |  |
| RT |  |  |
| no | 1.00 (Reference) | <.001*** |
| yes | 11.34 (4.79 ~ 26.84) |  |

Notes: *, two-sided P values <0.05; **, two-sided P values <0.01; ***, two-sided P values <0.001. Abbreviations: UAC, uterine cervical adenocarcinoma; DFS, disease-free survival; pT stage, pathological stage of primary tumor; pN stage, pathological stage of lymph nodes; pM stage, pathological stage of metastasis; FIGO, International Federation of Gynecology and Obstetrics; LVSI, lymph-vascular space invasion; ChT, chemotherapy; RT, radiotherapy; HR, hazard ratio; CI, confidence interval.
